# Finding functional associations between prokaryotic virus orthologous groups: a proof of concept

**DOI:** 10.1186/s12859-021-04343-w

**Published:** 2021-09-15

**Authors:** Nikolaos Pappas, Bas E. Dutilh

**Affiliations:** Theoretical Biology and Bioinformatics, Department of Biology, Science for Life, Utrecht University, Utrecht, The Netherlands

**Keywords:** Function prediction, Machine learning, Bacteriophages

## Abstract

**Background:**

The field of viromics has greatly benefited from recent developments in metagenomics, with significant efforts focusing on viral discovery. However, functional annotation of the increasing number of viral genomes is lagging behind. This is highlighted by the degree of annotation of the protein clusters in the prokaryotic Virus Orthologous Groups (pVOGs) database, with 83% of its current 9518 pVOGs having an unknown function.

**Results:**

In this study we describe a machine learning approach to explore potential functional associations between pVOGs. We measure seven genomic features and use them as input to a Random Forest classifier to predict protein–protein interactions between pairs of pVOGs. After systematic evaluation of the model’s performance on 10 different datasets, we obtained a predictor with a mean accuracy of 0.77 and Area Under Receiving Operation Characteristic (AUROC) score of 0.83. Its application to a set of 2,133,027 pVOG-pVOG interactions allowed us to predict 267,265 putative interactions with a reported probability greater than 0.65. At an expected false discovery rate of 0.27, we placed 95.6% of the previously unannotated pVOGs in a functional context, by predicting their interaction with a pVOG that is functionally annotated.

**Conclusions:**

We believe that this proof-of-concept methodology, wrapped in a reproducible and automated workflow, can represent a significant step towards obtaining a more complete picture of bacteriophage biology.

**Supplementary Information:**

The online version contains supplementary material available at 10.1186/s12859-021-04343-w.

## Background

The vast diversity across all environments of viruses that infect bacteria and archaea, herein together referred to as bacteriophages, has long been postulated [1]. Viral metagenomics or viromics, the application of metagenomics methods to identify and study viruses in mixed samples, has enabled us to more effectively catalogue bacteriophage diversity. New information is being accrued both on the level of their taxonomy and on the level of their genomic content and encoded functions. New lineages are being discovered in different environments, such as crAssphage [2] and megaphages [3] in the human gut or novel Vibrionaceae-infecting phages with relatively wide host-range in marine biomes [4], shedding light into the unexplored component of the virosphere’s diversity [5].

Concurrently, our view of the functional repertoire of phages is being expanded. Characterizing the functions encoded by bacteriophage proteins is an invaluable step towards understanding their role as drivers of processes within an ecosystem, via their interactions with their bacterial hosts. For example, it is becoming clear that bacteriophage genomes may encode functions that were previously thought to be carried out exclusively by cellular organisms, such as auxiliary metabolic genes involved in photosynthesis and carbon metabolism [6] or sulfur and nitrogen cycling [7].

However, functional annotation of most viral proteins remains challenging. Paez-Espino et al. [8] were able to match 5.1% out of a total of 6.1 million proteins to ones with a known function by using similarity searches of proteins against a constructed database of 25,000 viral protein families, while Elbehery et al. [9] were able to find matches for up to 50% for a relatively well-studied environment, the human gut. These examples demonstrate the shortcomings of classical approaches, such as sequence similarity searches, for the annotation of viral genes and proteins [10]. This is mainly because (1) currently deposited bacteriophage sequences only capture a small portion of their naturally occurring diversity, and (2) they exhibit a high mutation rate and higher frequency of novel genes, leading to higher sequence diversity.

Clustering the encoded proteins into protein families provides a framework for rapid function annotation [11], since typically proteins in the same family perform similar functions. A useful resource for bacteriophage protein families is the prokaryotic Virus Orthologous Groups (pVOGs) database [12], although we note that establishing orthologous relationships between proteins encoded by viruses can be challenging, as horizontal gene transfer and recombination between viral genomes is a major driver in their evolution. The pVOGs are based on nearly 300,000 protein-coding genes from approximately 3000 viruses infecting bacterial or archaeal hosts, that have been clustered into 9518 orthologous groups. However, currently 83% of the 9518 pVOGs consist of hypothetical proteins that do not have a meaningful functional annotation.

Biological function is a loosely defined term and can take different meanings depending on the context in which is examined. This gives rise to a framework that describes the function of a protein on the molecular, cellular or phenotypic level [13]. In comparative genomics, an established approach to overcome the issues arising from lack of homology-based evidence is using genomic information to improve function prediction [14]. In prokaryotes, genes encoding for functionally associated proteins often exhibit similar phylogenetic profiles i.e. co-occurrence patterns across several genomes [15]. Additionally, they tend to be commonly regulated and are organized in single transcriptional units (operons) with the same orientation [16]. Similar observations have been made for viral genomes, where genes are organized in cassette structures with preserved orientation [17].

Predicting functions for unknown genes and their products from their association with other genes, commonly referred to as guilt-by-association [18], can be an alternative to functionally annotate proteins. The notion of functional association has been successfully used for organisms from all domains of life in the popular STRING database [19]. It encompasses a great number of proteins that are functionally associated in comprehensive networks of interactions. A version of STRING specifically designed for viral proteins is currently available (Viruses.STRING, [20]). Its main focus is to catalogue virus-host interactions, expanding protein–protein interaction networks from within-species to cross-species interactions.

Here, we explore the potential of functional association between pairs of pVOGs by predicting their interaction based on guilt-by-association signals. We measured seven features on a reference set of bacteriophage genomes for pairs of pVOGs, namely co-occurrence, average genomic distance, orientation relationship (co-orientation, convergent, divergent), average nucleotide identity and average amino acid identity and integrated these values to predict pVOG-pVOG interactions by using a Random Forest classifier. Although we train the current version of the prediction pipeline with a relatively small dataset of known physically interacting protein pairs [21], we make the associated software publicly available so that users can apply it to larger datasets once they become available.

## Methods

### Interaction datasets

A discretely labeled ground truth dataset of interacting (1) and potentially non-interacting (0) protein pairs for supervised machine leaning with Random Forest [22] was constructed as follows: profile Hidden Markov Models (HMMs) of bacteriophage protein families and their functional annotations were retrieved from the pVOGs database [12] (http://dmk-brain.ecn.uiowa.edu/pVOGs/downloads.html, accessed 01/2020). To establish the interaction dataset (1) we used the IntAct database, a publicly available database of physical molecular interaction information [21] (accessed 04/2019) to define a positive set of 102 interacting protein pairs, labeled with 1. While IntAct contains protein pairs that were experimentally shown to engage in physical molecular interactions, this is not a requirement for our prediction pipeline and we note that the positive set may be readily expanded to include more loosely defined interaction pairs once they become available.

Non-interaction (0) is difficult to establish since interaction between protein pairs may depend on very specific cellular conditions. Thus, ten different negative sets were randomly sampled from all possible protein pairs that were present in RefSeq [23] bacteriophage genomes on which IntAct proteins were found, but that were not present in the positive set. Bacteriophage genomes were retrieved from the RefSeq database with the query.

‘Viruses[ORGN] NOT "cellular organisms"[ORGN] AND vhost bacteria[filter] OR vhost archaea[filter] AND "complete genome" [All fields]’

for viruses infecting bacteria and archaea (accessed on 01/2019). Protein–protein interactions were translated to pVOG-pVOG interactions using hmmsearch v3.2.1 with default options [24], querying all pVOG HMM profiles against the list of IntAct proteins and selecting the hit with the highest bitscore (Additional file 1).

As we were interested in predicting interaction between protein pairs on the same genome, all pairs that could not be significantly matched to pVOG pairs which co-occurred on at least one genome were excluded. From the remainder we randomly selected ten different negative (non-interacting) datasets containing 102 pVOG pairs each, which were each combined with the same 102 positive (interacting) pVOG pairs to form ten training datasets N1-N10. Finally, the target dataset consisted of all possible pairwise combinations of the 9518 pVOGs, excluding self-pairs and the 204 pairs from the ground truth set.

### Feature selection and measurement

A description of all measured genomic features is provided in Table 1. All bacteriophage genomes were 6-frame translated with the transeq utility from the EMBOSS package version 6.6.0.0, options “-clean-frame 6-table 11” [25]. An hmmsearch was subsequently carried out with all pVOGs HMM profiles against the translated RefSeq genomes and results were parsed with the help of custom python scripts to extract the relevant information about genomic occurrence, distance and orientation.

**Table 1:** Features used in this study for the prediction of pVOG-pVOG functional association

Name	Description
Co-occurrence	Calculated by dividing the number of genomes where both pVOGs have hits by the total number of genomes where either of the pVOGs have hits (Jaccard similarity)
Average distance	Minimum distance in nucleotides of the alignment envelopes (sensu hmmsearch) between the two pVOGs, averaged across all common genomes
Co-orientation	Fraction of hits where both pVOGs are found on the same strand
Convergent orientation	Fraction of hits where both pVOGs are found on opposite strands with 3’ ends facing each other
Divergent orientation	Fraction of hits where both pVOGs are found on opposite strands with 5’ ends facing each other
Mean ANI	Average Nucleotide Identity (ANI) of all genome pairs where the pVOGs co-occur, calculated with fastANI [26]
Mean AAI	Average Amino acid Identity (AAI) of all genome pairs where the pVOGs co-occur, calculated with CompareM [27]

All features except co-occurrence are calculated on genomes where both pVOGs have a significant hit

### Classification with random forest

Hyperparameter tuning was performed based on a split of each dataset to 70% training and 30% holdout. The training set was used for a randomized search and fivefold cross-validation approach available from python’s scikit-learn package version 0.21.3 [28]. A subset of the parameters known to affect the classifier’s performance were selected, such as the maximum depth and number of decision trees to use. A range of values was defined for these parameters and 500 classifiers were built based on a random selection of the whole parameter space. Each classifier was used for a fivefold cross-validation to select the model with the best combination of hyperparameters.

This process gave us a best model for each of the ten datasets. To calculate the performance of every model on different datasets, the remaining nine sets were used as input to the obtained model. The same split of the data to 70% training and 30% holdout was applied, but no hyperparameter optimization was performed. The final combination of model and training set for the classification of the target dataset was determined based on its consistent higher performance across the metrics described below.

### Performance evaluation, model and dataset selection

For every classification problem, there are four possible outcomes:

- An observation, in this case a pVOG-pVOG interaction, can be correctly identified and labeled as belonging to the positive class (True Positive, TP).
- An observation can be correctly identified and labeled as belonging to the negative class (True Negative, TN).
- An observation can be incorrectly identified and labeled as belonging to the positive class, while in reality it belongs to the negative class (False Positive, FP).
- An observation can be incorrectly identified and labeled as belonging to the negative class, while in reality it belongs to the positive class (False Negative, FN).

These can be summarized in various metrics for assessing the performance of the classification. Here, we used the following:

- *Accuracy*: The sum of correctly labeled interactions, either as positive or negative, divided by the sum of all predictions ((TP + TN)/(TP + TN + FP + FN)).
- *Precision*: The sum of true positives divided by the sum of all positive predictions. (TP/(TP + FP))
- *Recall* (or *sensitivity*): The sum of true positives divided by the sum of true positives and false negatives. ( TP / (TP + FN))
- *F1 score:* The harmonic mean between precision and recall. ((2 x (Precision + Recall))/(Precision + Recall))
- *Area Under the Receiver Operating Characteristic Curve (AUROC):* A single value representing the performance of the classifier, when taking the true positive and false positive rates into account. In general, an AUROC score higher than 0.5 is desired, which signifies that a classifier performs better than random [29].

### Annotation processing

The pVOGs remain among the most comprehensive functional annotation platforms for viral proteins. Currently, pVOGs are functionally annotated with all the terms of its constituent proteins [12]. As pVOGs contain different numbers of proteins and protein annotations are free text fields, these may vary both in number and in syntax format. All occurrences of “hypothetical protein” were replaced with “unknown”, and the words “protein” and “putative” were removed. After this reformatting the annotation with the highest count was selected as a single annotation describing the pVOG. All statistics referring to the annotations were calculated based on the processed annotations. To quantify the similarity between the functional annotations of pVOG pairs, a corpus was constructed from the terms appearing in the annotations of all pVOGs. The following terms were excluded: 'hypothetical', 'hypotheical', 'hypothetical-acquired', 'hypotthetical', 'hypothethical', 'hyphothetical', 'hypothetical-protein', 'hypho', 'predicted', 'protein', 'unknown', 'putative', 'phage', 'bacteriophage', 'no', 'annotation', 'provided', 'gene', 'and', 'in', 'conserved', '#', and '&'. Next, a weight was assigned to each word, based on its inverse frequency of appearance (1—frequency of term) to assign a higher weight to more unique terms. For each pVOG a frequency vector of its own annotation terms was constructed. Finally, we calculated the weighted cosine distance between the two term-frequency vectors of pVOG pairs that had at least three or ten terms each.

## Results

**Fig. 1:**
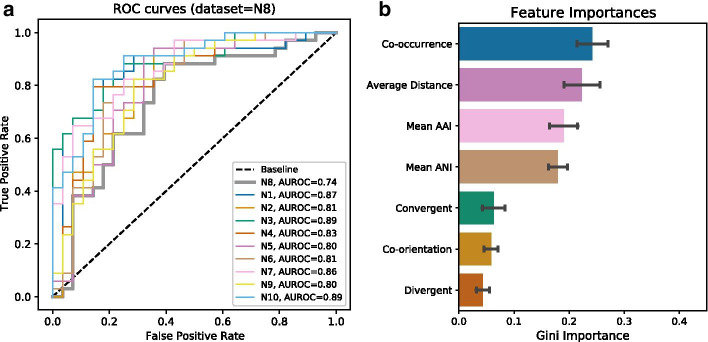
**a** ROC curves illustrating the performance of the final best Random Forest classifier on the dataset that was used for hyperparameter optimization (N8, thick gray line), as well as across the remaining datasets (AUROC = Area Under the Receiver Operating Characteristic). **b** Relative importance of each feature for classification, based on the Gini importance

We explored the potential of functional association of bacteriophage proteins, by predicting interactions between pairs of pVOGs. We evaluated the performance of several Random Forest classifiers across 10 different datasets N1–N10 (see Methods). First, each dataset was split into 70% training and 30% holdout sets. After hyperparameter optimization on 500 different classifiers, the classifier with the best performance on the holdout set was selected as a candidate model. Then, the remaining nine datasets were split into 70% training and 30% holdout sets. The training set was used to train the candidate model from before and to make predictions on the holdout set, providing us with performance metrics for each combination of model and dataset. This process was repeated for all datasets. We thus obtained a classifier, optimized based on dataset N8 and performing better than the rest of the nine candidate models across all datasets. It achieved a mean accuracy of 0.77 (± 0.03) with a mean AUROC score of 0.83 (± 0.05) (Fig. 1a; Additional files 2, 3, 4). In absolute numbers, 47.5 (± 1.9) out of 62 interactions in each holdout dataset were correctly classified either as positive or negative. Its mean precision was 0.78 (± 0.04) and the mean recall score 0.8 (± 0.05).

Feature importance scores were calculated using Gini importance, defined as the total decrease in node impurity averaged over all trees of the forest [30]. Intuitively, it gives a measure of how the accuracy of classification changes when the values of a feature are randomly permuted. The most important feature for predicting interaction between a pair of pVOGs was the co-occurrence between the putative interactors across bacteriophage genomes (mean relative importance 0.24 ± 0.02) (Fig. 1b). While this is expected because proteins need to be present on the same genome to interact, it is still a significant result because the signal might be reduced if the candidate proteins would frequently occur on different genomes. The average distance between the HMM hits on the genomes had the second highest relative importance (mean = 0.22 ± 0.03) followed by mean AAI (0.19 ± 0.03) and mean ANI (0.18 ± 0.02) between the genomes containing the hits. The genomic orientation features did not appear to play an important role in predicting protein interaction, possibly because many bacteriophage proteins tend to be encoded in the same direction [31] (Additional files 4, 5).

We applied the best performing classifier to the target dataset of all pVOG pairs passing our filtering criteria, i.e. co-occurring on at least one genome. This dataset includes 9369 out of the total 9518 unique pVOGs (98.4%). In total, 766,080 of the 2,133,027 pVOGs pairs (35.9%) were predicted to interact, with 443,786 positive interactions (57.9%) having a high confidence, based on a cutoff of ≥ 0.65 from 500 decision trees. Additional file 5 shows the distribution of interaction probabilities predicted by the Random Forest classifier for all 2,133,027 target pVOG pairs, showing that a known and unknown pVOG could be linked in many cases. Note that the cutoff of 0.65 between low- and high-confidence predictions is arbitrary, but stricter than the cutoff of ≥ 0.5 that is used in many classification studies using Random Forest.

Next, we leveraged information from the predicted interactions to provide a preliminary annotation for pVOGs with unknown functions. In total, 53,999 predicted interactions (7%) in the final dataset occurred between pVOG pairs where both were annotated. These interactions can be viewed as an additional means of validation of our method (Table 2). Furthermore, 325,464 predictions (42.4%) had one unannotated pVOG interacting with a pVOG with known functional annotation. For the remaining 386,617 interacting pairs (50.5%) neither of the pVOGs had an annotation.

**Table 2:** Top fifteen predicted interactions between pairs of pVOGs with annotated functions

pVOG A	pVOG B	P (interaction)	Annotation A	Annotation B
VOG5511	VOG6633	0.996	Tail fiber protein	Putative tail protein
VOG1215	VOG4545	0.996	Minor tail protein	Tape measure protein
VOG0796	VOG5106	0.996	Terminase small subunit	Phage terminase large subunit
VOG0205	VOG4586	0.996	Putative head taill joining protein	Major tail protein
VOG4553	VOG4773	0.994	Major capsid protein	Capsid portal protein Q
VOG4604	VOG5027	0.994	Portal protein	Head morphogenesis protein
VOG4565	VOG9941	0.994	Lysozyme	putative dna maturase b
VOG4555	VOG5106	0.994	Scaffolding protein	Phage terminase large subunit
VOG1190	VOG2368	0.994	Portal protein	Ribonucleoside triphosphate reductase, alpha chain
VOG4545	VOG9209	0.994	Tape measure protein	Minor tail protein L
VOG4545	VOG4599	0.994	Tape measure protein	Minor structural protein
VOG0641	VOG4763	0.994	Holin	Peptidase_S74 protein
VOG0641	VOG4763	0.994	Holin	Minor structural protein
VOG0692	VOG0796	0.994	Minor capsid protein	Terminase small subunit
VOG4811	VOG6163	0.994	Tape measure	Putative tape measure protein

*P (interaction)* represents the mean predicted probability of a pVOG pair to interact from 500 individual classifiers (decision trees) in the Random Forest. The full list is provided in Additional file 6

A total of 7627 out of the original 7974 pVOGs with unknown function (95.6%) were matched with to pVOGs with annotated functions when using a cutoff of 0.65, providing preliminary hints about their function through guilt-by-association (Additional file 2). Based on the confusion matrix, which was calculated from 62 holdout interactions in the final best model derived from the N8 dataset, TP = 27, FP = 10, TN = 18, and FN = 7. The false discovery rate FP/(TP + FP) of the final model was 0.27, hence we expect less than ~ 120 thousand false positive pairs among the 443,786 predicted functional associations.

**Fig. 2:**
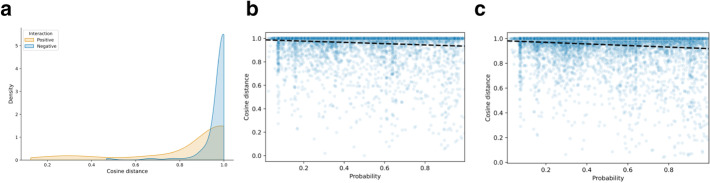
**a** Density plot of weighted cosine distance scores for the positive and negative interactions in the final training set (n = 184). **b** Linear regression plot of probability of interaction and weighted cosine distance for a set of 592,062 predictions between pVOG pairs with at least three informative annotation terms each (r = − 0.126, *p* = 0.0). **c** Same as (b), for a set of 146,456 predictions between pVOG pairs with at least ten informative annotation terms each (r = − 0.141, *p* = 0.0)

We explored the relationship between the similarity in annotated functions of two pVOGs and their predicted interaction probability. To quantify similarity in annotated functions we used a weighted cosine distance between the annotation term vectors (see Methods), where pVOGs with similar functional annotations have a low cosine distance value and vice versa. We confirmed this method by testing it on the final training set, where we observed that the weighted cosine distances of interacting pairs were lower than of non-interacting ones (Fig. 2a). As expected, the separation between interacting and non-interacting protein pairs was imperfect, reflecting a noisy signal. Figure 2b and c show the correlation between predicted pVOG-pVOG interactions with a minimum of three and ten annotation terms, respectively. Notwithstanding the noisy signal, we observed an inverse relationship between the cosine distance and the probability of interaction, providing further support for our proof-of-concept approach to finding functional associations between viral proteins. Notably, pVOG pairs with a very low predicted interaction score all have a high cosine distance, while the majority of pVOG pairs with a very low cosine distance, especially the well-annotated ones with at least ten annotation terms, tend to have a high predicted interaction score.

## Discussion

High throughput viromics experiments are shining new light on environmental bacteriophages, arguably the most unexplored components of the biosphere. Although sequence assembly now allows these phages to be mapped at genomic resolution, understanding the functions of their encoded proteins remains challenging. Here, we developed a method to integrate diverse genomic signals to predict functional associations between bacteriophage proteins, through a machine learning approach, thus providing initial leads for their interpretation. The classifier performed well on the holdout datasets, with the best model predicting 27 of 34 positive interactions and 18 of 28 negative interactions correctly (Fig. 1a). In our analyses, the co-occurrence and average distance between two genes were identified as the most important features, consistent with the existence of genomic organization [32] and functional gene cassettes [18] in bacteriophages. Interestingly, orientation is not important, consistent with transcriptional directionality being more uniform in bacteriophages than in bacteria [31].

Several developments may be expected to further improve the interaction predictions. First, we used a small ground truth set limited to only 102 positive interactions, representing physically interacting proteins in the IntAct database [21]. Application of the Random Forest classifier allowed us to predict interaction probability between millions of protein pairs, demonstrating the utility of machine learning approaches for datasets where limited information is available. However, we expect that future expansion of the training dataset with a larger number of high-quality known interactions will almost certainly increase the accuracy of the predictor. Second, additional meaningful guilt-by-association features may be included into the predictor (Table 1). For example, gene co-expression provides a strong functional signal that is complementary to the genomic signals included here [33, 34]. Third, the use of a larger reference set of viral genomes should also be beneficial, as it will better reflect any genomic signals that link interacting phage proteins. Moreover, including diverse viral sequences, including those from metagenomic datasets will allow functional associations between proteins to be identified in a greater diversity of viruses, decreasing database bias [35]. We expect that the automated, reproducible snakemake-based [36] workflow provided through the GitHub repository (see Methods) will help users to readily implement these and other additions and further improve the prediction of functional associations between bacteriophage proteins.

## Conclusions

To conclude, we predicted functional associations for 95.6% of the phage protein families (pVOGs) that were previously not functionally annotated, by predicting their interaction with functionally annotated proteins. At an expected false discovery rate of 0.27, this still represents a significant step towards obtaining a more complete picture of bacteriophage biology. Approaches such as the one described here, will greatly benefit the ongoing efforts of bacteriophage genome annotation and, by extension, will facilitate ecological and evolutionary inferences about their role in shaping microbial communities.

## Supplementary Information

**Additional file 1**. Mapping of interacting RefSeq protein pairs to their best matching pVOGs with the reported E-value and bit score

**Additional file 2: Fig. S1**. Performance metrics for 10 different RF classifiers obtained from all datasets (N1–N10). Each classifier was optimized with the respective dataset and performance was evaluated using the remaining nine datasets as input. Boxplots show the median, lower and upper quartile with the whiskers extending to 1.5 times the interquartile range; the diamonds are outliers. Y-axis starts from 0.4 for visualization purposes

**Additional file 3: Fig. S2**. Mean absolute accuracy of all classifiers. Barplots represent the mean number of correct classifications; error bars represent standard deviation. Red dashed line: number of interactions in the holdout set (n =62)

**Additional file 4: Fig. S3**. ROC curves for the 10 datasets (N1–N10) used for performance evaluation. Each dataset was used as a ground truth set for parameter optimization of a Random Forest classifier (70% training). The resulting best model was used for predictions on the holdout set (30% of the original) and its ROC curve is depicted by a thicker line. The remaining 9 datasets were used as input for the best model obtained for training (70%) and holdout (30%) and their ROC curves are shown as more transparent lines (AUROC = Area Under the Receiver Operating Characteristic)

**Additional file 5: Fig. S4**. Distribution plot of prediction probabilities for the 2,133,027 pVOG pairs in the target dataset. Positive interactions have a probability greater than 0.5. Stacked bars are colored based on the annotation status of the pVOGs according to the legend

**Additional file 6**. Main table containing all processed pVOG pairs, their predicted label (0 for negative, 1 for positive), prediction probability, available annotations (raw and processed) and features values. Accessible via https://zenodo.org/record/4576466

## Data Availability

Raw data are provided on the publicly accessible data sharing platform Zenodo https://zenodo.org/record/4576599. Source code used for the analyses is available on https://github.com/MGXlab/pvogs_function. A snakemake [36] workflow, wrapping all necessary steps in an automated fashion is also available on the GitHub repository.

## Abbreviations

AAI: average amino acid identity
ANI: average nucleotide identity
AUROC: area under the receiver operating characteristic
FN: false negative
FP: false positive
HMM: hidden Markov model
pVOG: prokaryotic virus orthologous group
TN: true negative
TP: true positive
