# Supplementary figures and images for: Finding functional associations between prokaryotic virus orthologous groups: a proof of concept
Source: BMC Bioinformatics. 2021 Sep 15;22:438. doi: 10.1186/s12859-021-04343-w (PMC8442406; doi:10.1186/s12859-021-04343-w)

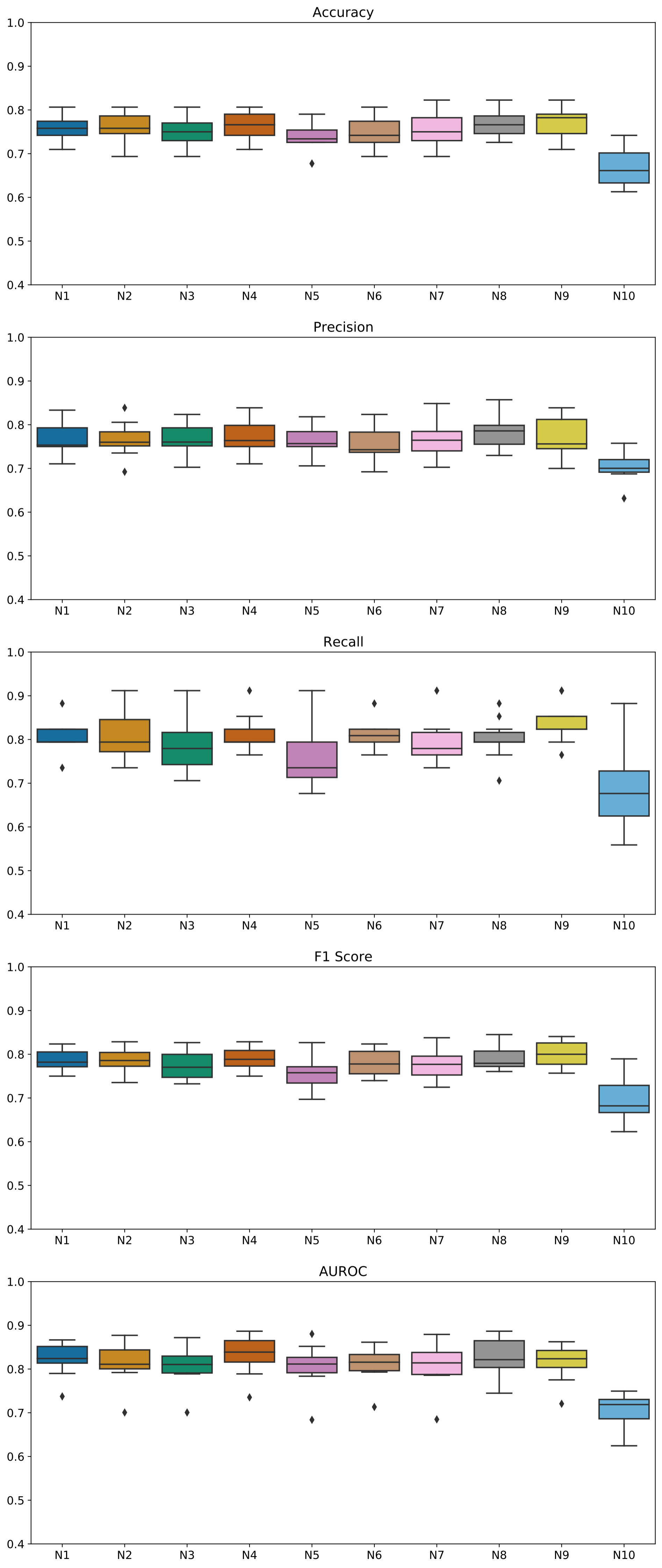

Supplement: Supplementary file 2 — Additional file 2: Fig. S1. Performance metrics for 10 different RF classifiers obtained from all datasets (N1–N10). Each classifier was optimized with the respective dataset and performance was evaluated using the remaining nine datasets as input. Boxplots show the median, lower and upper quartile with the whiskers extending to 1.5 times the interquartile range; the diamonds are outliers. Y-axis starts from 0.4 for visualization purposes [file 12859_2021_4343_MOESM2_ESM.pdf]

Absolute accuracy

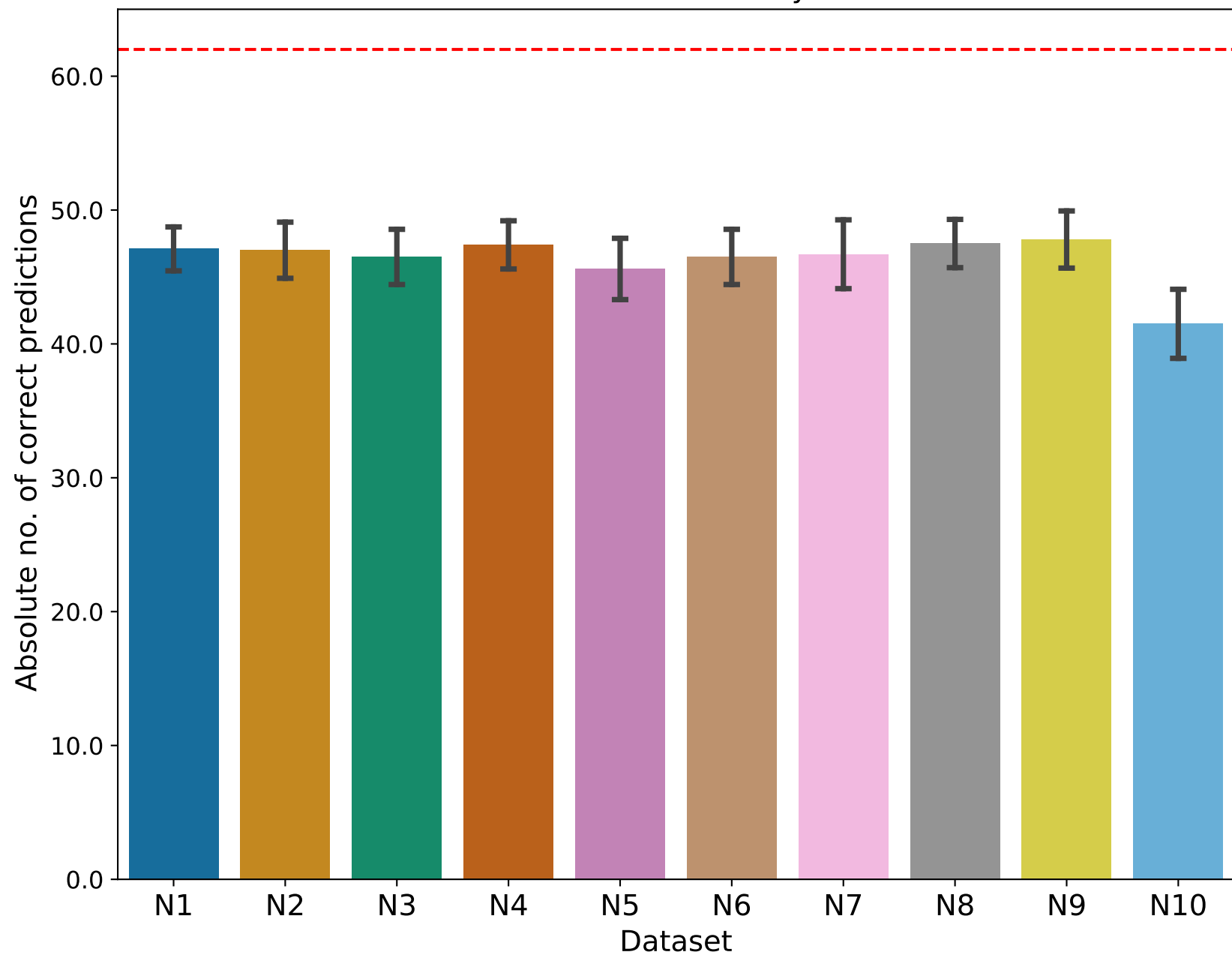

Supplement: Supplementary file 3 — Additional file 3: Fig. S2. Mean absolute accuracy of all classifiers. Barplots represent the mean number of correct classifications; error bars represent standard deviation. Red dashed line: number of interactions in the holdout set (n =62) [file 12859_2021_4343_MOESM3_ESM.pdf]

ROC Curves for all datasets

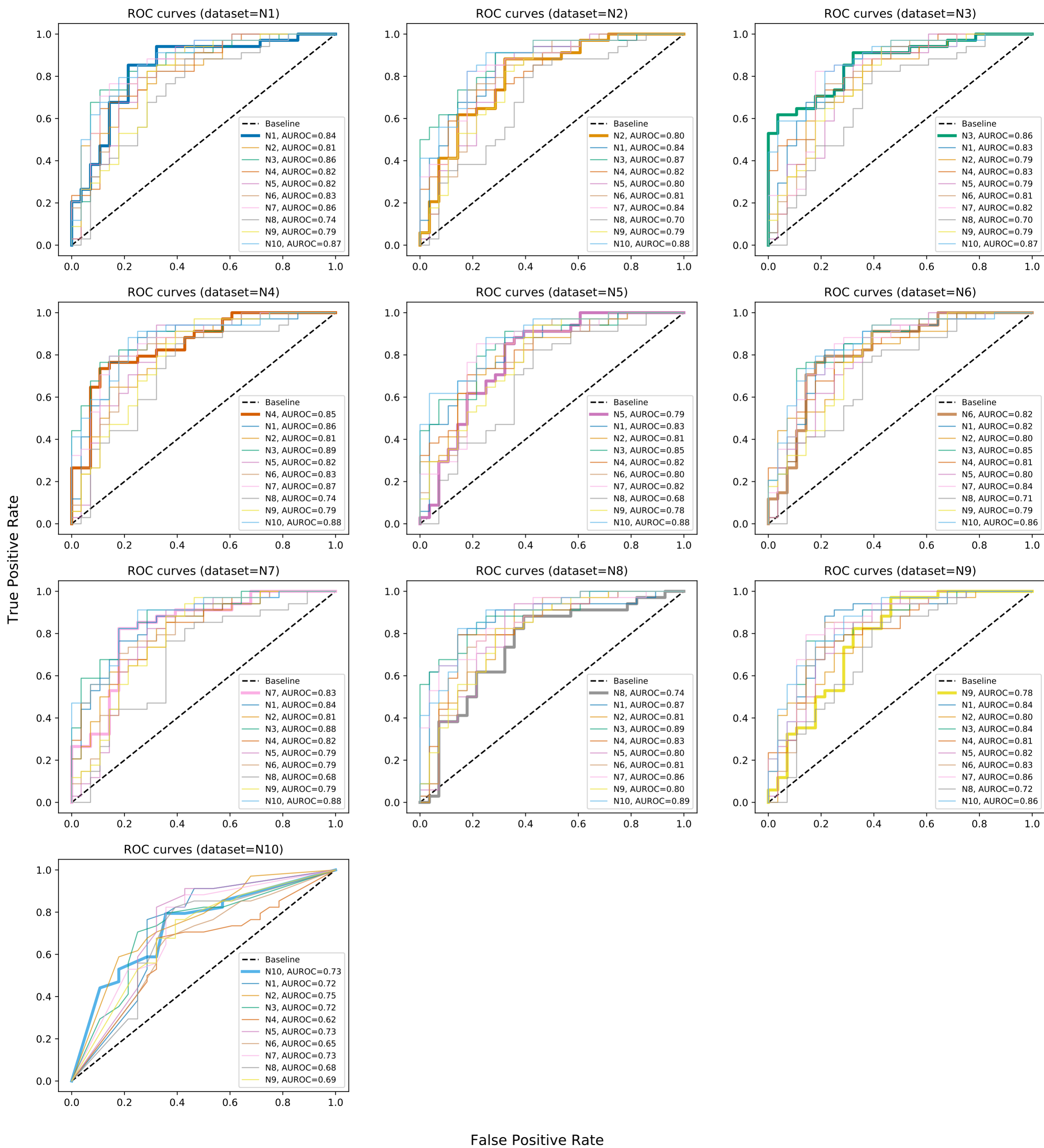

Supplement: Supplementary file 4 — Additional file 4: Fig. S3. ROC curves for the 10 datasets (N1–N10) used for performance evaluation. Each dataset was used as a ground truth set for parameter optimization of a Random Forest classifier (70% training). The resulting best model was used for predictions on the holdout set (30% of the original) and its ROC curve is depicted by a thicker line. The remaining 9 datasets were used as input for the best model obtained for training (70%) and holdout (30%) and their ROC curves are shown as more transparent lines (AUROC = Area Under the Receiver Operating Characteristic) [file 12859_2021_4343_MOESM4_ESM.pdf]

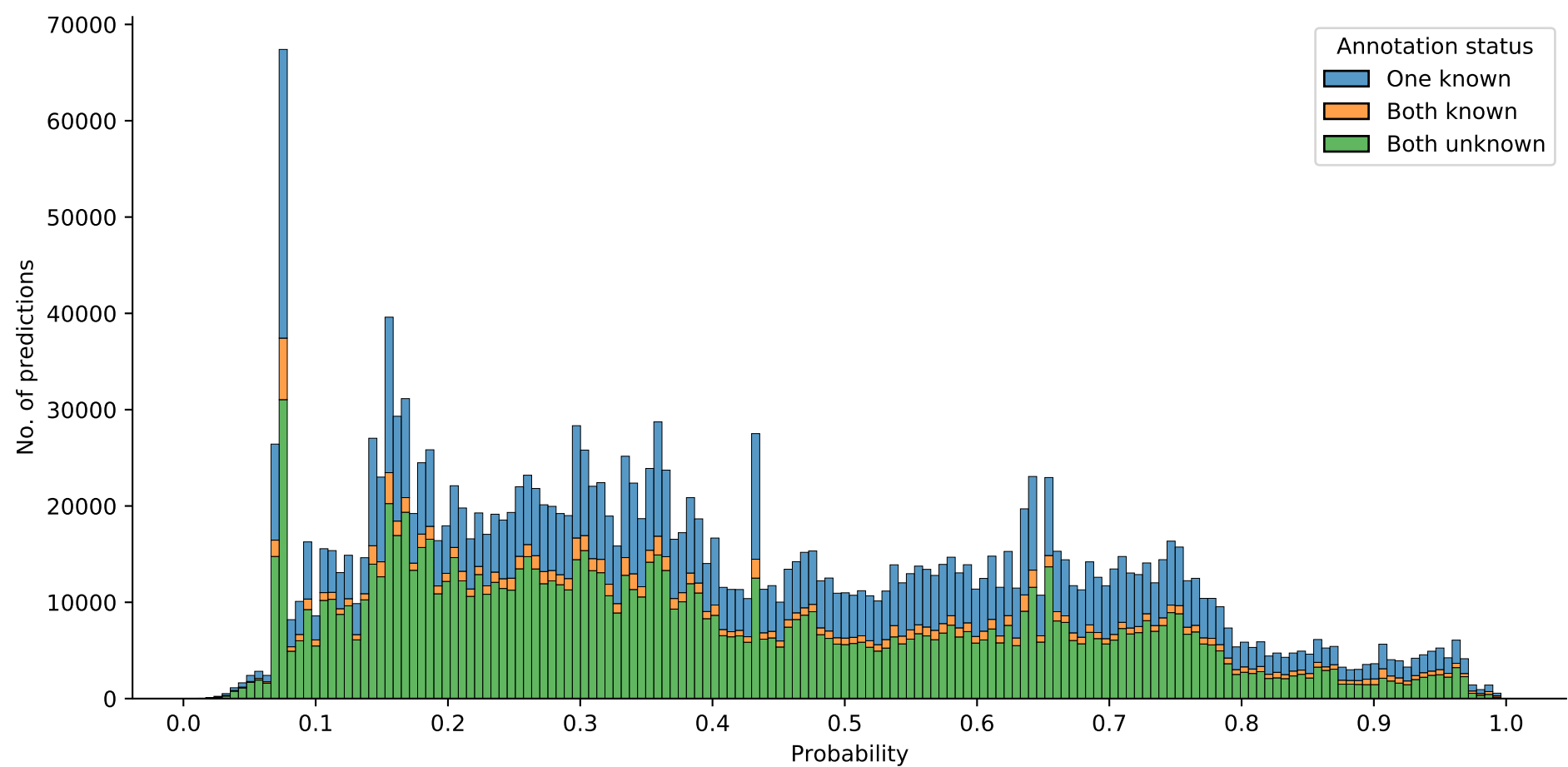

Supplement: Supplementary file 5 — Additional file 5: Fig. S4. Distribution plot of prediction probabilities for the 2,133,027 pVOG pairs in the target dataset. Positive interactions have a probability greater than 0.5. Stacked bars are colored based on the annotation status of the pVOGs according to the legend [file 12859_2021_4343_MOESM5_ESM.pdf]
